# Policy Decisions and Use of Information Technology to Fight Coronavirus Disease, Taiwan

**DOI:** 10.3201/eid2607.200574

**Published:** 2020-07

**Authors:** Cheryl Lin, Wendy E. Braund, John Auerbach, Jih-Haw Chou, Ju-Hsiu Teng, Pikuei Tu, Jewel Mullen

**Affiliations:** Duke University, Durham, North Carolina, USA (C. Lin, P. Tu); University of Pittsburgh, Pittsburgh, Pennsylvania, USA (W.E. Braund);; Trust for America’s Health, Washington, DC, USA (J. Auerbach);; Taiwan Centers for Disease Control, Taipei, Taiwan (J.-H. Chou, J.-H. Teng);; University of Texas, Austin, Texas, USA (J. Mullen)

**Keywords:** health policy, public health, emerging infectious diseases, communicable diseases, epidemic, outbreak, universal health care, national health insurance, pneumonia, quarantine, data sharing, electronic medical records, respiratory infections, severe acute respiratory syndrome coronavirus 2, SARS-CoV-2, SARS, COVID-19, 2019 novel coronavirus disease, Taiwan, zoonoses, viruses, coronavirus

## Abstract

Because of its proximity to and frequent travelers to and from China, Taiwan faces complex challenges in preventing coronavirus disease (COVID-19). As soon as China reported the unidentified outbreak to the World Health Organization on December 31, 2019, Taiwan assembled a taskforce and began health checks onboard flights from Wuhan. Taiwan’s rapid implementation of disease prevention measures helped detect and isolate the country’s first COVID-19 case on January 20, 2020. Laboratories in Taiwan developed 4-hour test kits and isolated 2 strains of the coronavirus before February. Taiwan effectively delayed and contained community transmission by leveraging experience from the 2003 severe acute respiratory syndrome outbreak, prevalent public awareness, a robust public health network, support from healthcare industries, cross-departmental collaborations, and advanced information technology capacity. We analyze use of the National Health Insurance database and critical policy decisions made by Taiwan’s government during the first 50 days of the COVID-19 outbreak.

On December 31, 2019, China reported 27 cases of an unidentified viral pneumonia outbreak in Wuhan, Hubei Province, to the World Health Organization (WHO; *1*). During the 2003 outbreak of severe acute respiratory syndrome (SARS), Taiwan experienced 346 cases and 37 deaths (*2*). Considering >8,000 cases of SARS occurred globally in 2003 and the outbreak claimed 774 lives worldwide, the Taiwan government was particularly cautious about the emerging infectious disease in nearby China in 2019. Taiwan’s proximity to China and the frequency of travelers between the 2 countries created concern the virus could spread. Because millions of Taiwan citizens living and studying in China were expected to return home for the January 11 presidential election and impending Lunar New Year holiday and Taiwan is a favorite destination for tourists from China (*3*), intensified public anxiety warranted heightened disease prevention measures.

Informed by lessons from the 2003 SARS outbreak, Taiwan had systems in place to fight the potential new epidemic. The country has a robust nationwide public health network, comprehensive universal healthcare for all citizens, vibrant medical research and pharmaceutical industries, and improved infection control practices. We delineate and analyze the critical policy decisions and cross-departmental collaborations in the Taiwan government and Taiwan Centers for Disease Control (Taiwan CDC) during the first 50 days of the COVID-19 epidemic. Of note, the centralized, real-time database of the country’s National Health Insurance (NHI) helped support disease surveillance and case detection. Taiwan CDC’s comprehensive response and innovative use of the NHI database effectively delayed and contained community transmission in the country, even as the number of confirmed cases surged in neighboring countries in Asia starting in mid-February.

## Devising and Updating Travel and Disease Control Policies

While most of the world was preparing for the 2020 New Year, Taiwan CDC began health screening of passengers on flights arriving from Wuhan. Within a week, the government assembled a cross-departmental taskforce and an expert team of leaders in infectious diseases, public health, and laboratory sciences. The government raised the travel advisory to Wuhan to level I–watch and alerted the healthcare community to report to Taiwan CDC on patients with respiratory symptoms and fever or presumptive pneumonia who had recently traveled to Wuhan. At the same time, the Taiwan CDC epidemiology laboratory started developing and producing test kits adapted from existing diagnostic modalities for pneumonia of unknown etiology.

As Taiwan CDC took the lead, public and private healthcare providers, local governments, and health departments looked to the central government for guidance regarding preparedness and response. The country quickly updated infection control practices and strategies established during the 2003 SARS epidemic, such as installation of infrared temperature checkpoints and border quarantine at airports and seaports. Following Taiwan CDC’s outbreak prevention guidelines, hospitals swiftly instituted screening booths to monitor the temperature of persons entering the facility, offer hand sanitizer, and separate persons with fever or related ailments. In addition, Taiwan increased stockpiles of personal protective equipment (PPE) for healthcare workers, predesignated potential isolation wings and hospitals, and created a daily nationwide inventory of available intensive care and negative-pressure isolation rooms, including the number that could be refitted when needed.

On January 15, 2020, Taiwan CDC classified the novel coronavirus as a class-V communicable disease, which institutes legal measures, including mandated reporting and quarantine. For instance, under class-V, healthcare providers are required by law to report suspected cases to Taiwan CDC within 24 hours, and the government can isolate or quarantine persons confirmed or suspected to be infected at designated sites. The Wuhan travel advisory was elevated to level II–alert the next day and later to level III–warning (Figure; Appendix Table). Reporting criteria were broadened to include persons showing symptoms who had not traveled to China recently but had close contact with persons who had confirmed or suspected cases. In addition, specimen testing parameters were expanded. On January 20, Taiwan activated its Central Epidemic Command Center (CECC), which is equivalent to an Emergency Operations Center in the United States.

**Figure:**
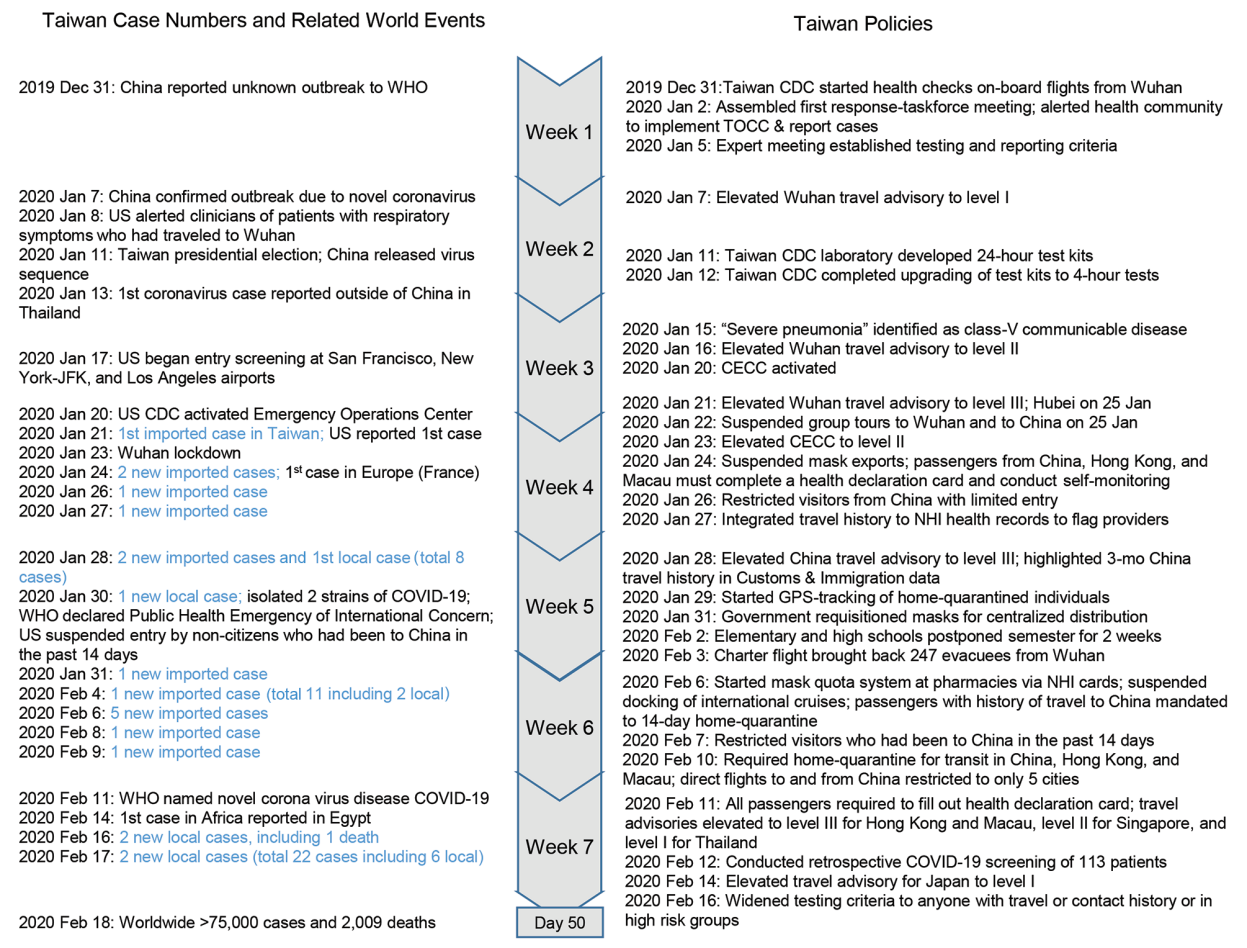
Timeline of policy decisions during the first 50 days of COVID-19, Taiwan. Blue text indicates cases in Taiwan. Information collected from Taiwan CDC, CDC, and WHO. Because of differences in global time zones, some events might be recorded or announced with 1-day discrepancy in different reports, news, and publications. CDC, US Centers for Disease Control and Prevention; CECC, Central Epidemic Command Center; COVID-19, 2019 novel coronavirus disease; NHI, National Health Insurance; Taiwan CDC, Taiwan Centers for Disease Control and Prevention; TOCC, travel, occupation, contact, and cluster; WHO, World Health Organization.

### Border Quarantine

Border quarantine procedures are managed by staff from regional offices of Taiwan CDC stationed at airports and seaports. Staff screen all incoming passengers by using no-touch, video-recordable infrared thermometers, which were installed during the 2003 SARS outbreak. Staff also monitor passengers for specific symptoms, provide timely health education, and conduct health evaluations, including sample collection or testing, as needed. In addition, staff report suspected cases to the centralized database of Taiwan CDC and to local health departments for follow-up monitoring or care and refer or transport symptomatic persons to hospitals according to infectious disease regulations, when needed.

Beginning December 31, 2019, Taiwan CDC implemented enhanced border quarantine measures, which included temporary onboard health checks on persons arriving on flights from Wuhan. As the outbreak spread internationally, in late January 2020, Taiwan began requiring passengers to manually or electronically complete a health declaration card detailing any symptoms or diseases, and travel and contact histories for case investigation or contact tracing, if necessary. In addition, Taiwan CDC staff determined the need and gave instructions for self-monitoring or home quarantine, depending on current policies and any special situations.

### Case Detection

The enhanced border quarantine procedures led to early detection of a suspected case of COVID-19. On January 20, a 55-year-old woman reported fever, cough, and shortness of breath at her airport health screening upon arrival from Wuhan. She was transported directly to the hospital, averting local exposure. She reported that she wore a mask and remained in her seat for the duration of the flight. The crew and other passengers, who had no prolonged direct interaction with her, passed the health evaluation at the airport and were directed to complete a 14-day self-monitoring regimen at home. During self-monitoring, passengers and crew were required to record their temperature twice daily, stay home, or wear a mask if they had to go out; as an extra measure, they had to respond to daily telephone checks by infectious disease staff.

On January 21, the passenger with symptoms was confirmed to have COVID-19, the first known imported case in Taiwan. The same day, the United States announced its first case in a 35-year-old man who had returned from Wuhan on January 15 and was later admitted to a hospital in Washington State on January 19 (*4*,*5*).

With confirmed cases reaching 1,400 globally, including cases in Europe (*6*), Taiwan’s disease investigation teams worked through the week-long Lunar New Year holiday. Beginning on January 24, Lunar New Year’s Eve, all passengers traveling from China, Hong Kong, and Macau were required to complete a health declaration card and travel history upon arrival in Taiwan. Arriving passengers were given instructions for self-monitoring and a phone number for inquiries or concerns; this procedure was later expanded to cover arrivals from all destinations. Passengers from Wuhan and Hubei Province and persons who had close contact with confirmed cases were mandated to a 14-day home quarantine. Quarantine involved self-isolation without going out or having visitors, recording temperature and symptoms twice daily, and if living with others, wearing a mask at all times and taking precautions with household members.

To support Taiwan CDC’s surveillance, local civil offices were given the contact information of all home-quarantined persons in their jurisdiction. Local health department personnel or district administrators familiar with the communities conducted daily telephone checks on these home-quarantined persons in their areas. Persons who were not compliant with home quarantine orders were turned over to law enforcement and tracked by police officers. Repeat offenders could be fined or confined to designated facilities.

As the number of persons on home isolation in Taiwan grew to tens of thousands, GPS functionality and cameras on personal or government-dispatched smartphones were used for monitoring and case identification. Recognizing the challenges of the need for seemingly healthy persons to stay home for 2 weeks, miss work and school, and avoid outside contacts, local governments set up quarantine-care centers to provide support and counseling, which strengthened the barrier against potential community transmission. Staff in PPE could conduct home visits, arrange meal deliveries, and bring essential supplies to persons living alone to help them comply with the quarantine order. A 24-hour public epidemic hot line was opened for questions or reporting. Taiwan CDC upgraded its interactive mobile phone application, Disease-Prevention Butler, and supplemented it with an artificial intelligence chatbot to provide accurate, timely information and gather concerns for analysis and response.

Group tours from Taiwan to China were suspended, and tours from China and residents of Hubei were banned. All citizens from China were later banned from entry into Taiwan, with few exceptions (Appendix Table). For groups already in Taiwan at the time of border quarantine, tour leaders were required to conduct and report daily health checks of their members. Students enrolled in Taiwan colleges or universities who had gone home to China for the winter break and holiday were asked to postpone their return to Taiwan for 2 weeks; those who arrived early were self-quarantined in separate dormitories.

On January 30, WHO declared a public health emergency of international concern and urged international coordination to investigate and control the spread of COVID-19 (*7*). Confirmed cases climbed to >7,800 globally; Taiwan had 9, including 1 case of local transmission in a man infected by his wife who returned from Wuhan (*8*).

### Information Technology and Cross-Departmental Cooperation

Other government agencies in Taiwan also contributed expertise and increased capacity during the crisis. Taiwan CECC partnered with civil and law enforcement departments for quarantine monitoring, as described. In addition, the CECC asked the NHI to integrate recent history of travel to China from the database of Customs and Immigration to supplement the NHI’s centralized cloud-based health records. After Customs and Immigration data were integrated, the NHI system flagged records so medical providers would be aware of patients’ travel history when they made an appointment or came in. Later, all confirmed and suspected case contacts reported to Taiwan CDC also were added to the NHI database.

Because all providers are required to submit claims to the single-payer platform within 24 hours, the comprehensive NHI database had near–real-time information that let clinicians and Taiwan CDC track or trace back all doctor visits. The NHI patient records included complete health history, underlying health conditions, and recent progression of symptoms, treatments, and hospitalization related to respiratory syndrome. These data helped pinpoint high-risk patients and persons likely to have had contact with infected cases. In addition, the NHI database gave Taiwan CDC the ability to quickly identify new patterns of symptoms or clustered cases and the source or path of infection. The high security and privacy policy of the NHI information technology system permitted data sharing only for purposes of combatting the epidemic and was restricted to 1-way transmission of specific information from other departments to the NHI database. No health records or other personal information were available to anyone outside of the health system.

The Customs and Immigration database also displayed warnings about travel history to Wuhan and China within the previous 3 months so border control staff could identify persons who had been to the COVID-19 epicenter for additional health screening. The Ministry of Foreign Affairs negotiated and coordinated the evacuation of Taiwan citizens stranded in Wuhan after the city went into lockdown on January 23 (*9*) and, later, those who were passengers onboard the Diamond Princess cruise ship docked in quarantine off the coast of Japan (*10*). The Ministry of Transportation managed charter flight arrangements, and the special biohazard cadets from the Ministry of Defense were called to help disinfect the planes and affected airport areas afterwards. Repatriated citizens and cruise ship passengers went through health screenings before boarding airplanes and were immediately tested for COVID-19 upon arrival in Taiwan. One person evacuated from Wuhan tested positive for the coronavirus and was directly transported to a hospital. All others passed a double-negative criterion, having 2 negative test results 24 hours apart, and went to a government-managed quarantine facility for 14 days, where they received check-ups 3 times a day. No subsequent cases manifested.

### Social Norms and Mask Shortages

After the 2003 SARS outbreak, persons in Taiwan, Japan, and several other countries in Asia began wearing medical face masks during influenza season or in crowded public spaces, such as on subways (*11*). Wearing a mask also is considered good practice for persons with a cold, and persons with allergies or a weakened immune system are expected to wear a mask (*12*). Therefore, many citizens had supplies at home or rushed to acquire masks once the epidemic was announced, despite Taiwan CDC advising that healthy persons did not need a mask, except when visiting hospitals or crowded, enclosed places.

Anticipating a surge in demand, Taiwan’s prime minister suspended mask exportation at the end of January. News of shortages soon emerged in different parts of the world, partially attributed to the delayed and reduced exports from China, the largest mask-producing country in the world, because dozens of cities in China were on lockdown and demand increased in the country (*13*,*14*). The Taiwan government requisitioned domestically made medical and surgical masks and invested to quickly expand production. To accomplish better distribution across the population, Taiwan introduced a temporary rationing system. Every resident’s NHI card, which is already linked to thousands of pharmacies and hundreds of local health centers nationwide, became their identification to obtain masks in their neighborhood. In addition, a government-funded, mobile phone application (Mask Finder, https://mask.pdis.nat.gov.tw), developed through a public-private partnership, helped citizens locate supply distribution points and showed updates on availability. Health promotion messages on indications for wearing a mask and handwashing routine were widely disseminated in all media.

### Clinical and Pharmaceutical Research Capacity and Case Investigation

Starting in early January 2020, the Taiwan CDC laboratory began developing real-time reverse transcription PCR (RT-PCR) diagnostic protocols by leveraging previous experience sequencing SARS and Middle East respiratory syndrome coronaviruses. China released the full genomic sequence of the novel coronavirus on January 11, and by January 12, the Taiwan laboratory team introduced an upgraded, 4-hour test kit, shortened from the initial 24-hour test. The upgraded test had a high sensitivity of 10–100 copies/reaction, which is comparable to the standard assays recommended by WHO. The laboratory staff continued to accelerate testing speed and capacity, developing the ability to test >1,100 samples/day. By the end of February, Taiwan was able to test 2,450 samples/day by using public and select contracted private laboratories.

In late January, 2 strains of the coronavirus were successfully isolated by a university and a government-funded research institute in Taiwan. Research and development of drugs, vaccines, and a rapid testing kit continued, some through public-private or international partnerships.

As it became known that persons could have COVID-19 and have mild or no symptoms, no travel history, or no definitive case contact (*15*), Taiwan CDC further widened its testing and reporting criteria to minimize local transmission. At the time, only 3 cases of local transmission had been identified, all contracted from family members with recent travel history. To improve case detection, on February 12, Taiwan CDC conceived a retrospective COVID-19 screening scheme. The screening encompassed persons who had tested negative for influenza in the previous 14 days but who reported having severe influenza complications, were under surveillance for upper respiratory symptoms, were part of a cluster of influenza cases, or received a diagnosis of pneumonia but did not respond well to treatment. Using the NHI database, the team pinpointed 113 suspected patients, 1 of whom, case 19 in Taiwan, tested positive for COVID-19 on February 15 and died that evening. This discovery triggered the required confirmed-case contact investigation, which located and tested dozens of the patient’s family members and close contacts. The patient’s asymptomatic brother tested positive on the same day, and 2 more family members with minor symptoms tested positive in the next 2 days. Other close contacts tested negative but were stipulated to a 14-day home quarantine, and hundreds more possible contacts were put on self-monitoring for 2 weeks. The source of infection for case 19 later was identified by using collaborative triangulation of multiple departments’ databases and disease investigation and traced to a passenger who returned from China. Without retrospective screening and access to the comprehensive NHI database, such cases would have gone undetected.

On day 50 of the global epidemic, February 18, WHO reported >75,000 cases and >2,000 deaths worldwide (*16*). Among the 22 cases confirmed in Taiwan, local transmissions were limited to 5, primarily between family members. Despite a credible international report that modeled outbreak dynamics and predicted Taiwan would have the second highest case importation outside of China (*17*), early prevention measures, stringent border control, and aggressive efforts to combat community spread have continued to be effective as of March 2020.

## Policy Implications

With the outlook of COVID-19 still unclear, health authorities around the world continue to be on high alert. Since February 2020, the Taiwan government and CECC have focused more on detecting and isolating local cases to contain potential local spread, while maintaining and updating travel restrictions to limit foreign entry from highly affected areas. The experience of SARS generated instrumental lessons in disease control measures and policy planning for government agencies and hospitals in Taiwan. It also improved the public’s health behavior and hygiene practices, such as increased uptake of influenza and other vaccinations, frequent handwashing, and use of hand sanitizers and masks (*12*,*18*–*20*). In addition, the 2003 SARS outbreak had heightened infection transmission awareness and provided better mental preparedness for the new pandemic. Timely, clear communication with the public also has fostered trust and built community capacity for the public to partner with the government in containment and mitigation.

During any health crisis, a robust health system is crucial to support the surge of medical care and testing needed (*21*). Taiwan has a solid public health, medical, and insurance infrastructure distributed throughout the country. This infrastructure consists of local health departments and centers staffed by healthcare professionals trusted by local residents, particularly in the rural areas where private practices are scarce; hospitals, medical centers, and clinics that strongly support a well-coordinated infectious disease network for preparedness and response; and a comprehensive NHI that covers >99% of the population with high-quality providers and low out-of-pocket cost. The interconnected health system reduces barriers to doctor appointments and follow-up visits, which helped capture suspected cases with minor symptoms. Furthermore, the single-payer NHI model affords centralized health records of population-level longitudinal data and the capability of merging information from other government databases. This connectivity proved a valuable tool for analysis and case investigation during disease outbreaks, including dengue, influenza, SARS, and the current COVID-19 pandemic.

Interagency collaboration, data sharing, and timely mobilization of human capital and resources are equally vital to a response (*22*). Taiwan followed WHO standards on testing and case definition and shared updated disease information and virus sequences on International Health Regulations (https://www.who.int/ihr/en) and other global health platforms. With CECC’s authority to coordinate works across departments and enlist additional personnel during an emergency, Taiwan CDC has been capable of handling the growing volume of regular and new tasks.

In addition, the legislature approved emergency funding to ensure disease control efforts did not fall short and to mitigate the economic effects of the outbreak. The funding included compensating lost wages for persons working part-time or without paid sick leave during the quarantine. Compensation also permitted time off for persons with children or elderly family members who were sick or had contact with confirmed cases. These incentives, modeled after actions taken during the 2003 SARS outbreak, aided in isolation compliance.

Unlike SARS, in which patients were only infectious when febrile (*23*), persons with COVID-19 could have no or minimal symptoms, remain undiagnosed but contagious, and pose a greater threat of local transmissions (*24*). As the pandemic evolves, global cases likely will increase because of community spread, expanded laboratory capacity, and wider testing criteria. The timing, locations, and policies of travel advisories and entry restrictions, in addition to testing and reporting criteria, are critical to epidemic control but vary across countries. From a public health perspective, recognizing the ideal time to institute or terminate these policies and measuring their effectiveness can be challenging.

## Conclusions

Taiwan’s robust public health and healthcare systems, combined with public acceptance of protective policies influenced by the 2003 SARS outbreak, likely bolstered efficient implementation of policies in the first 50 days of the COVID-19 outbreak. At the same time, Taiwan’s response to COVID-19 might have overshadowed other health threats, such as seasonal influenza and chronic diseases. Strategic prioritization of other public health functions and resources and broader government operations will be necessary. As the outbreak continues, Taiwan will need to evaluate associated policy decisions to sustain the system.

Taiwan built on lessons learned from SARS, and some of the successful strategies during the current pandemic could inform policy approaches by other governments. In countries that rely heavily on state and local actions, intergovernmental and interjurisdictional coordination and adequate funding are needed to assure emergency preparedness and response capacity. An integrated approach that incorporates public health, human services, and healthcare systems can increase resilience and better prepare nations for future events.

Appendix Additional information on policy decisions in response to 2019 novel coronavirus diseases, Taiwan.
