# Supplementary material for: Policy Decisions and Use of Information Technology to Fight Coronavirus Disease, Taiwan
Source: Emerg Infect Dis. 2020 Jul;26(7):1506–12. doi: 10.3201/eid2607.200574 (PMC7323533; doi:10.3201/eid2607.200574)
Supplement: Appendix — Additional information on policy decisions in response to 2019 novel coronavirus diseases, Taiwan. [file 20-0574-Techapp-s1.pdf]

Article DOI: https://doi.org/10.3201/eid2607.200574

Policy Decisions and Use of Information Technology to Fight COVID-19, Taiwan

Appendix

Appendix Table. Timeline of 2019 novel coronavirus infectious disease incidents and policies in Taiwan and the world, December 31, 2019–February 23, 2020*

Date†	Taiwan	Globally
2019 Dec 31	Taiwan CDC activated border infectious disease response, started on-board health check for flights from Wuhan	China: reported to WHO on 27 cases of unknown pneumonia in Wuhan, Hubei Province
2020 Jan 2	First taskforce response team meeting;<br>Alerted providers to inquire and report suspected cases	
2020 Jan 7	Raised Wuhan travel advisory to level I-watch	China: confirmed outbreak was associated with novel coronavirus
2020 Jan 8		USA: alerted clinicians to look out for patients with respiratory symptoms and known travel to Wuhan
2020 Jan 10		China: 41 cases
2020 Jan 11	Taiwan CDC developed 24-h test kit<br>Presidential election	China: released virus sequence; 1 reported death
2020 Jan 12	Taiwan CDC improved test kit to 4-h testing time	WHO named 2019 novel coronavirus 2019-nCov
2020 Jan 13	2 infectious disease experts visited Wuhan Jan 13–14	Thailand: 1 reported case and the 1 case reported outside of China
2020 Jan 15	Taiwan CDC classified the “severe specified pneumonia” as Class-5 communicable disease	Japan: 1 reported case
2020 Jan 16	Elevated Wuhan travel advisory to level II–alert	China: 45 cases
2020 Jan 17		USA: CDC began entry screening at San Francisco, New York John F. Kennedy, and Los Angeles International Airports
2020 Jan 20	Activated Central Epidemic Command Center (CECC)	US CDC activated Emergency Operations Center<br>China: 291 cases in 4 provinces and cities<br>South Korea: 1 reported case
2020 Jan 21	First confirmed imported case in Taiwan;<br>Elevated Wuhan travel advisory to level III–warning	China: 440 cases<br>Outside China: 5 cases in 5 countries<br>USA: first reported case‡; raised travel advisory to Wuhan to level-II
2020 Jan 22	Suspended tour groups to Wuhan	China: 571 cases;<br>Outside China: 8 cases<br>Hong Kong: 1 reported case
2020 Jan 23	Elevated CECC to level-II	China: 830 cases; 1 reported death outside of Hubei; Wuhan lockdown<br>Outside China: 14 cases in 9 countries<br>Vietnam: 2 reported cases, 1 reported transmission outside of China<br>Singapore: 1 reported case
2020 Jan 24	Taiwan: 3 cases, 2 new imported cases; required all passengers from China, Hong Kong, Macau to complete Health Declaration Card and 14-d self-monitoring; Executive Yuan suspended mask export for 1 month (later extended)	China: 1,287 cases; Beijing and Shanghai on highest level of public health emergency; Shanghai Disney closed<br>Outside China: 24 cases in 11 countries<br>Europe: 1 reported case in France<br>USA: second reported case in Chicago
1/25/20	Lunar New Year; suspended all tour groups to China; elevated Hubei travel advisory to level III, others level II	China: 1,975 cases in 30 provinces and cities;<br>Outside China: 37 cases in 14 countries<br>Malaysia: 1 reported case<br>Australia: 1 reported case
2020 Jan 26	Taiwan: 4 cases, 1 new imported case; restricted visitors from China with limited entry; postponed students returning from China for 2 wk after holiday	USA: 5 cases
2020 Jan 27	Taiwan: 5 cases, 1 new imported case; passengers from Hubei required 14-d home-quarantine; added	USA: CDC issued updated travel advisory for China to level III

Date†	Taiwan	Globally
	Hubei travel history to National Health Insurance's health records to flag providers	
2020 Jan 28	Taiwan: 8 cases, 2 new imported cases and first local spousal transmission; elevated travel advisory to China to level III; noted Wuhan travel history to Customs and Immigration records to flag border control	Germany: 1 reported case<br>Japan: classified 2019 novel coronavirus as "designated infectious disease"
2020 Jan 29		USA: government evacuated 201 American citizens, mostly diplomats, out of Wuhan via Anchorage;<br>Japan: first plane evacuation
2020 Jan 30	Taiwan: 9 cases, 1 new local spousal transmission	Geneva: WHO declares Public Health Emergency of International Concern<br>China: 7,736 cases, 170 deaths<br>Outside China: 7,818 cases in 19 countries<br>USA: first reported person-to-person transmission in Illinois<br>Tibet: 1 reported case<br>India: 1 reported case<br>Philippines: 1 reported case
2020 Jan 31	Taiwan: 10 cases, 1 new imported case of total 8; requisition of domestic masks for centralized distribution; universities and research institute successfully isolated 2 COVID-19 virus strains	Outside China: 114 cases in 22 countries<br>USA: Department of Health and Human Services declares Public Health Emergency; President Trump announced suspension of entry by non-citizens who been to China in the past 14 d;<br>Italy: 2 cases in tourists from China<br>United Kingdom: England reports 1 case<br>Russia: 1 reported case
2020 Feb 1		China: 14,380 cases<br>Outside China: 158 cases in 26 countries<br>Philippines: 1 reported death and first reported outside of China
2020 Feb 2	Postponed primary and secondary schools for 2 weeks until Feb 25	China: second city lockdown in Wenzhou on the eastern coast<br>USA: suspended entry by foreigners who had been to China in the past 14 d
2020 Feb 3	A charter flight evacuated 247 Taiwan citizens from Wuhan<br>Updated reporting: dual testing of flu and COVID-19	
2020 Feb 4	Taiwan: 11 cases, 1 new case in an evacuee from Wuhan; suspended docking of cruises carrying confirmed or suspected cases within 28 d or docked in China, Hong Kong, or Macau within 14 d	Hong Kong: 1 reported death<br>Japan: Diamond Princess cruise ship docked in Yokohama and started 14-d quarantine onboard
2020 Feb 5		China: >24,000 cases<br>Hong Kong: all passengers entering from China required 14-d quarantine
2020 Feb 6	Taiwan: 16 cases, 5 new imported cases; suspended entry of all nationals from China, including Hong Kong and Macau; Taiwan citizens arriving from China, Hong Kong, and Macau required 14-d home-quarantine; suspended international cruises from docking; introduced mask quota	Singapore: 30 cases<br>Italy: 3 cases
2020 Feb 7	Suspended entry by foreigners who had been to China in the past 14 d	China: whistleblower Dr. Li Wenliang dies<br>USA: ≈800 citizens evaluated from Wuhan
2020 Feb 8	Taiwan: 17 cases, 1 new of total 15 imported cases; Aquarius cruise docked at Keelung, tested 128 suspected cases including crew members, all were negative and all 1,709 passengers required 14-d self-monitoring	Global: 805 deaths, surpassing 774 deaths from SARS<br>Canada: 213 citizens evacuated from Wuhan arrive for 14-d quarantine in Ontario<br>France: announced 2 schools shut down next week
2020 Feb 9	Taiwan: 18 cases, 1 new imported case	China: >37,000 cases
2020 Feb 10	Passengers transited in Hong Kong or Macau required 14-d home-quarantine; suspended direct passenger flights to and from China except 5 cities	China: 42,638 cases, 1,016 deaths<br>Outside China: 443 cases
2020 Feb 11	All arrivals required to complete Health Declaration Card including travel and contact history of past 14 d; elevated Hong Kong and Macau travel advisory to level-III; suspended entry of Hong Kong and Macau residents with few exceptions including students, required self-quarantine	WHO named the illness caused by novel coronavirus disease COVID-19 and the virus severe acute respiratory syndrome coronavirus-2 (SARS-CoV-2)<br>Hong Kong: 42 cases
2020 Feb 12	Initiated retrospective screening	

Date†	Taiwan	Globally
2020 Feb 14	Elevated Japan travel advisory to level I; opened call center to counsel persons under home or mandatory quarantine	China: 66,492 cases<br>Outside China: 589 cases in 28 countries<br>African: 1 case in Egypt
2020 Feb 15	Taiwan: 20 cases, 16 imported, 2 new local cases of brothers identified from retrospective investigation;<br>first death in case 19	1 death in Europe and outside of Asia in France, a traveler from Wuhan<br>Japan: 1 death
2020 Feb 16	Broadened testing pool for enhanced community surveillance	US citizens evacuated from Diamond Princess cruise ship returned to California and Texas for 14-d quarantine
2020 Feb 17	Taiwan: 22 cases, 2 new cases in family of case19;<br>247 Taiwan citizens evacuated from Wuhan completed 14-d quarantine, only 1 confirmed case still hospitalized	USA: 15 cases in 7 states<br>Japan: 66 cases<br>Hong Kong: 58 cases<br>Macau: 10 cases<br>Global: >71,000 and 1,775 deaths; 156 cases in 14 countries outside of China
2020 Feb 18	Day 50<br>Taiwan: 22 total confirmed cases, including 16 imported cases and 1 death	China: 74,185 cases;<br>Global: >75,000 total cases and 2,009 deaths<br>Outside China: 965 cases, 5 deaths<br>Singapore: 81 cases<br>Hong Kong: 62 cases, 1 death<br>South Korea: hospital emergency department lockdown due to nosocomial infection<br>USA: evacuees from first Wuhan flight completed 14-d quarantine in California
2020 Feb 19	Taiwan: 24 cases; 2 new local cases, including a sister of case 19	China: 74,576 cases;<br>Outside China: 1,079 cases globally<br>South Korea: 46 cases; 15 new cases
2020 Feb 20	Identified infection source of case 19 after testing 248 contacts; elevated South Korea travel advisory to level-I	South Korea: 104 cases, 1 death<br>Global: 2,126 deaths
2020 Feb 21	19 Diamond Princess cruise passengers returned to Taiwan, all tested negative upon arrival, required 14-d group quarantine	China: 76,288 cases<br>Totals: >77,600 cases and >2,200 deaths<br>Outside China: 1,335 cases<br>USA: 34 cases, 13 local<br>Italy: 17 cases and 1 local transmission<br>South Korea: 204 cases, 4× the no. reported Feb 19<br>Iran: 18 cases, 4 deaths
2020 Feb 22	Diamond Princess passengers all tested negative for second test; elevated Japan and South Korea travel advisories to level-II	
2020 Feb 23	Elevated Italy and Iran travel advisories to level-I	China: 77,150 cases<br>Outside China: 1,974 cases globally

*Information collected from Taiwan Centers for Disease Control and Prevention (Taiwan CDC), US Centers for Disease Control and Prevention (CDC), World Health Organization (WHO), and CNN News. COVID, novel coronavirus infectious disease; SARS-CoV-2, severe acute respiratory syndrome coronavirus 2.

†Because of time differences around the world, some events may be recorded or announced with 1-day discrepancy in different reports, news, and publications.

‡US Centers for Disease Control and Prevention. Press release: First travel-related case of 2019 novel coronavirus detected in United States. 2020 Jan 21 [cited 27 Feb 2020]. https://www.cdc.gov/media/releases/2020/p0121-novel-coronavirus-travel-case.html
